# Pilot study of a culturally adapted psychoeducation (CaPE) intervention for bipolar disorder in Pakistan

**DOI:** 10.1186/s40345-017-0074-8

**Published:** 2017-02-11

**Authors:** Muhammad Ishrat Husain, Imran B. Chaudhry, Raza R. Rahman, Munir M. Hamirani, Nasir Mehmood, Peter M. Haddad, John Hodsoll, Allan H. Young, Farooq Naeem, Nusrat Husain

**Affiliations:** 1 Camden and Islington NHS Foundation Trust, St Pancras Hospital, 4 St Pancras Way, London, NW1 0PE UK; 2 Pakistan Institute of Living and Learning, Karachi, Sindh Pakistan; 3 Department of Psychiatry, Dow University of Health Sciences, Karachi, Pakistan; 4 Department of Psychiatry, Abbasi Shaheed Hospital, Karachi, Pakistan; 5 University of Manchester, Oxford Road, Manchester, UK; 6 Centre for Affective Disorders, Institute of PsychiatryPsychology and Neuroscience, King’s College London, London, UK; 7 Department of Psychiatry, Queens’ University, Kingston, ON Canada

**Keywords:** Bipolar disorder, Psychoeducation, Cultural psychiatry, Pakistan

## Abstract

**Background:**

Despite the use of maintenance medication, recurrence rates in bipolar affective disorder (BPAD) are high. To date, there are no clinical trials that have investigated the use of psychological interventions in bipolar disorder in Pakistan.

**Aim:**

The purpose of the study was to assess the feasibility and acceptability of a culturally adapted bipolar psychoeducation programme (CaPE) in Pakistan.

**Methods:**

Thirty-four euthymic bipolar I and II outpatients were randomized to either 12 weekly sessions of individual psychoeducation plus Treatment As Usual (Intervention) or Treatment As Usual (TAU) (Control). Outcomes were assessed using the Young Mania Rating Scale (YMRS), Beck Depression Inventory (BDI), EuroQoL (EQ-5D), Bipolar Knowledge and Attitudes and Questionnaire (BKAQ), and a self-reported measure of medication adherence (Morisky Medication Adherence Scale-4 items, MMAS-4). Effect sizes were derived from baseline adjusted standardized regression coefficients.

**Results:**

Retention in the study was good, 80% of patients in the TAU follow-up assessment and 100% of patients in the CaPE group attended all 12 sessions. Patient satisfaction was higher in the CaPE group relative to control (ES = 1.41). Further, there were large effect sizes shown for CaPE versus TAU for medication adherence (MMAS-4: ES = 0.81), knowledge and attitudes towards bipolar (BKAQ: ES = 0.68), mania (YMRS: ES = 1.18), depression (BDI: ES = 1.17) and quality of life measures (EQ-5D: ES ⇒ 0.88).

**Conclusions:**

Culturally adapted psychoeducation intervention is acceptable and feasible, and can be effective in improving mood symptoms and knowledge and attitudes to BPAD when compared with TAU. Larger scale studies are needed to confirm our findings.

*Trial registration*. Clinicaltrials.gov identifier NCT02210390

**Electronic supplementary material:**

The online version of this article (doi:10.1186/s40345-017-0074-8) contains supplementary material, which is available to authorized users.

## Background

Bipolar disorder (BD) is a significant cause of disability across the world (Wittchen et al. 2011; Vos et al. 2012). It is a common disorder, and for most sufferers, is highly recurrent and associated with significant psychosocial impairment as well as a high rate of completed suicide (Fajutrao et al. 2009) .

Long-term, or maintenance, pharmacological treatment with mood stabilizers, such as lithium, and specific antipsychotic drugs play an important role in the treatment of bipolar disorder. Despite the use of maintenance drug treatment, recurrence rates of bipolar disorder are high; a recent meta-analysis of long-term naturalistic studies reported a mean risk of at least one new syndromal BD episode of 55.2% (26.3%/year) with clinically determined treatments (Vázquez et al. 2015). This may partly reflect medication non-adherence. Two large Veterans Administration (VA) studies reported that approximately 50% of bipolar patients miss ≥20% of prescribed maintenance medication (Sajatovic et al. 2006, 2007). The limited efficacy of pharmacotherapy in bipolar disorder (Thase and Sachs 2000; Tohen et al. 2002) has increased the interest in the potential of adjunctive psychosocial interventions to enhance mood stability of patients in the long term. Studies suggest that the addition of non-pharmacological strategies helps not only in adherence to pharmacological treatment but can also improve patient’s psychological functioning in relapse prevention (Scott et al. 2007; Salcedo et al. 2016). A recent meta-analysis suggests that pharmacotherapy plus psychological intervention may significantly reduce recurrence rates and hospital admissions (Oud et al. 2016).

A variety of psychological interventions exist for bipolar disorder, including psychoeducation, CBT (Cognitive Behaviour Therapy), family focussed therapy and interpersonal and social rhythm therapy. The distinction between these different therapies is far from precise, and they share some common features, including provision of psychoeducation. Bearing this in mind, several guidelines recommend psychoeducation (PE) as the first-line choice of psychological intervention in bipolar disorder (Goodwin et al. 2016; Yatham et al. 2013). PE is a psychosocial approach that views bipolar disorder as a medical condition that would most greatly be benefited by education about the condition which empowers patients to play a larger role in their treatment. PE for bipolar disorder is aimed at improving medication adherence and also includes strategies to enhance awareness of triggers and associated problem solving strategies. PE encourages patients to be active participants in their own treatment and is often delivered in a group format (Stafford and Colom 2013). Structured PE programmes usually include the provision of information about the recurrence rate of the illness, medication and its adverse effects, triggering factors, the importance of adherence to treatment, how to manage symptoms, stress management, the risk of suicide, relevance of pregnancy, stigmatization, recognition of early recurrence symptoms, the avoidance of use of alcohol and other substances and the importance of leading a well-structured life (Colom et al. 2003). PE can be delivered in a group setting or on an individual basis and its proponents argue that it is a relatively straightforward and cost effective intervention (Scott et al. 2009; Yatham et al. 2013; Bond and Anderson 2015).

There are only a few studies investigating psychoeducation for bipolar disorder from low- and middle-income studies and these report conflicting findings (Javadpour et al. 2013; Cardoso Tde et al. 2014; Gumus et al. 2015).

## Objectives

The primary objective of this study was to pilot a 12-week individual culturally-adapted psychoeducational intervention (CaPE) to determine its’ feasibility in terms of recruitment, retention, adherence to the intervention and patient satisfaction with overall care. Secondary objectives included determining the efficacy of the CaPE intervention on improvement of knowledge and attitudes towards bipolar disorder, adherence to medication, and improvement of mood symptoms and quality of life.

## Methods

### Research design

The study was conducted between June 2012 and May 2015, and participants were recruited from outpatient psychiatric clinics of teaching hospitals in Karachi, Pakistan. A total of 34 participants were recruited to the pilot study and randomly allocated into two arms.; Intervention (individual Culturally adapted Psychoeducation, CaPE) and Treatment-As-Usual (TAU). Previous studies have recommended that sample sizes between 24 and 50 are sufficient for the purposes of a pilot trial (Julious 2005; Sim and Lewis 2012).

An off-site statistician carried out randomization using a web software (http://www.randomisation.com). The participants were followed up by a research assistant who completed baseline assessments and follow-up at 12 weeks (end of intervention).

### Inclusion and exclusion criteria

The inclusion criteria were: Diagnosis of DSM IV bipolar affective disorder, currently euthymic (BDI < 12 and YMRS < 8), age 18–65 years, participants engaged with the mental health services for preceding 6 months, able to give written informed consent, resident of the trial catchment area, and ability to speak Urdu/Punjabi/English.

The exclusion criteria were as follows: Severe cognitive impairment, currently experiencing relapse (mania, hypomania, mixed or depressive), being actively suicidal, the presence of any comorbid psychiatric illness such as substance misuse or alcohol dependence, according to DSM IV criteria.

### Recruitment of participants

In the first instance, the research clinician approached the clinical teams to inform them about the research study and the inclusion and exclusion criteria. The diagnosis of bipolar disorder was established by the patients’ regular outpatient psychiatrist based on DSM IV criteria. If patients met the entry criteria and were clinically stable and the clinical team agreed that the patient could be a possible participant, they introduced the study to the patient. With the patient’s agreement, the research clinician then approached the patient to explain the research study verbally and to provide them with the participant information sheet. Those patients who were confirmed to be eligible for the study and agreed to take part provided written informed consent and were recruited to the study.

### Interventions

#### Psychoeducation

This intervention consisted of 12 psychoeducation sessions, one session per week, that were administered on an individual patient basis, added to treatment as usual. Each session lasted for approximately 1 h, beginning with a 20–30 min presentation on the topic of the day, followed by a related exercise (e.g., drawing a life chart or compiling a list of potential triggers for relapse). Content was a reduced and modified version of the Barcelona Psychoeducation Program for bipolar disorders (Colom et al. 2003). The content of the sessions is summarized in Table 1. All sessions were provided by a Masters level clinical psychologist. The same psychologist provided each of the 12 sessions to ensure continuity. This psychologist received regular weekly supervision from authors NH and FN to ensure fidelity to the manual. Therapy sessions were recorded and tapes were randomly selected by the supervisor. Permission was obtained to translate and adapt the Barcelona programme for the purposes of the study. Baseline and follow-up ratings were conducted by blind raters.

**Table 1:** Sessions of the Culturally adapted Psychoeducation (CaPE) intervention

Session 1: Concept and causes
Session 2: Symptoms: Mania, Hypomania, Depression and mixed conditions
Session 3: Evolution and prognosis, psychoactive substance misuse
Session 4: Treatment with medication (Mood stabilizers, antipsychotics, antidepressants)
Session 5: Alternative therapies
Session 6: Risks associated with interruption of treatment
Session 7: Learning to detect early symptoms of relapse
Sesison 8: What to do when a relapse is detected?
Sesion 9: Regularity of habits
Session 10: Stress-control
Session 11: Prolem-solving strategies
Session 12: Final session

Cultural factors played a role in the decision to use individual psychoeducation in this study, since mental illness remains a source of stigma, particularly within Pakistani society. There is low awareness about the causes of mental illness in Pakistan (Waqas et al. 2014) which, in combination with low literacy rates, poor socioeconomic conditions and complex belief systems regarding black magic, possession and Jinn (demons) leads to public stigma. We opted to use individual psychoeducation due to a concern that patients may not be willing to discuss their personal difficulties in a group setting which in turn could lead to a high dropout rate.

#### Cultural adaptation of the intervention

Our group has culturally adapted interventions for depression using mixed methods, in Pakistan and the UK (Gater et al. 2010; Husain et al. 2014; Masood et al. 2015; Naeem et al. 2015a, b, c). The framework used considered three areas: assessment and engagement, awareness of cultural factors and adjustments in implementation (Naeem et al. 2015a, b, c). Most cultural adaptations of psychological treatments tend to be for implementation of the treatments rather than their content (Chowdhary et al. 2014), and we used the same principle for the CaPE intervention. For the purposes of the CaPE intervention, we used culturally acceptable idioms when explaining the concept, causes and symptoms of bipolar disorder (sessions 1–3) while taking into account participants’ lay perceptions on causes and treatment of mental illness. We also used local folk stories and images (e.g., to explain the concept of multiple perspectives) as well as examples from religious teachings. We incorporated simple strategies to improve engagement, which have worked in the past. These included speaking in the native language (Urdu) using culturally appropriate terms instead of jargon, and establishing a good rapport and a trusting relationship during the session. If the patient agreed, we also involved the main carer and families in the session, particularly for sessions 6, 7, and 8 (risks of treatment discontinuation and detection and management of relapse).

#### Treatment-as-usual

This group of patients received routine treatment, which in Pakistan means attending the outpatient clinic and taking prescribed medication.

## Outcome measures

The primary outcome of the study was the feasibility of the intervention in terms of recruitment and retention rates and patient satisfaction with overall care. Acceptance of the intervention was assessed using data on attendance and drop out. Patient satisfaction was assessed using a Visual Analogue Scale (VAS).

Secondary outcome measures included knowledge and attitudes towards bipolar disorder measured by using a questionnaire developed for use in primary care (BKAQ, see Additional file 1). Morisky 4-item self-reported measure of medication adherence (MMAS-4) (Morisky et al. 1986) was used to measure adherence to prescribed medication. Severity of mood symptoms was measured using the Young Mania Rating Scale (YMRS) (Young et al. 1978) (score of ≥20 indicating manic relapse), or Beck’s Depression Inventory (BDI) (Beck et al. 1961) (score of ≥14 indicating depressive relapse). Health-related quality of life was also measured using EuroQoL (EQ-5D) (Brooks and EuroQol Group 1996). All assessment scales were translated for use in Urdu and have previously been used in Pakistan (Farooq et al. 2010, Hashmi et al. 2007; Husain et al. 2014). The BDI has been assessed as valid for use in Pakistan however there is no evidence in the current literature regarding the cross-cultural validity of the YMRS (Ahmer et al. 2007; Husain et al. 2014; Khan et al. 2015).

All outcomes were measured at baseline and at week 12 (end of CaPE intervention).

### Statistical analysis

The aim of the analysis was to describe the feasibility and acceptability of the trial and CaPE intervention and estimate effect sizes for group differences in attitude, adherence, clinical and quality of life outcomes. For the EQ-5D two aspects of the EQ-5D were used, the 5 rating dimensions were combined into a weighted score with 1 indicating perfect health and 0 death (Brooks and EuroQol Group 1996). For the main analysis the UK weighting scheme was used with weighting for Thailand considered as a sensitivity analysis. Effect sizes were derived from standardized regression coefficients in which treatment group differences, adjusted for outcomes scores at baseline, were divided by the standard deviation of the outcomes. Following Cohen’s guidelines, effect sizes were treated as small (ES = 0.2), moderate (ES = 0.5) or large (ES = 0.8). As a pilot study, the sample size did not give sufficient power to reject null hypotheses; however, we derived inferential statistics on the intention to treat principle. Continuous outcome scores were analysed using ANCOVA models in which the mean group difference was adjusted for baseline outcome scores. As sample size was small and regression residuals not normally distributed we used bootstrap resampling (2000 iterations) to derive standard errors and bias corrected accelerated confidence intervals. Binary outcomes were analysed using Chi square or Fisher’s exact test if expected values were small. All statistical tests were two-sided and confidence interval level set at 95%. STATA 14.1 (StataCorp) was used to analyse the data.

## Results

There was little difference in socio-demographic variables between the two randomized groups, and this difference was not found to be statistically significant. 61.1% of participants in the CaPE group and 62.5% in the TAU group were male. There were proportionally more single persons in the TAU group (69%) than in the CaPE (39%) group. There was more unemployment in TAU (63%) than CaPE (50%). In the CaPE group, 22.2% or participants had over three previous hospital admissions compared to 37.5% of the TAU group (Table 2).

**Fig. 1:**
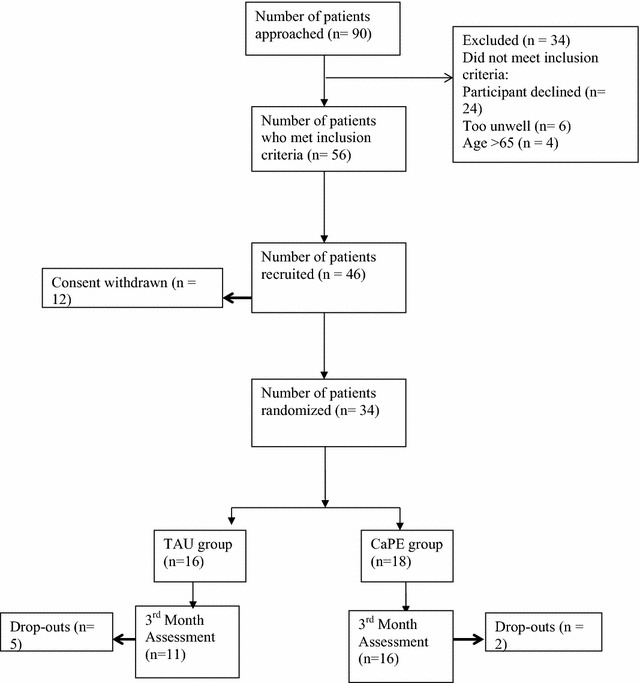
CONSORT diagram showing trial progress

Figure 1 summarizes participant flow through the study. 90 people were identified as suitable for recruitment. Of the 90 patients approached, 56 met the inclusion criteria. Of these 56 patients, 46 were recruited into the trial with 12 not willing to participate. 34 patients were subsequently randomized.

Of the 34 patients randomized, all were taking some form of prescribed psychotropic medication. 22 participants were taking a mood stabilizer (i.e., Lithium, Sodium Valproate, Lamotrigine) and 22 participants were taking an antipsychotic medication. 9 participants within the sample were taking an antidepressant and 7 were taking a benzodiazepine.

**Table 2:** Demographic characteristics of participants

	TAU (*N* = 16)	CaPE (*N* = 18)	*p* value
Age: median (IQR)	34.5 (27.5–43.5)	34.5 (28–40)	0.89
Gender			
Male: *n* (%)	10 (62.5)	11 (61.1)	1.00
Marital status			
Single: *n* (%)	11 (68.8)	7 (38.9)	0.18
Married: *n* (%)	6 (31.3)	10 (55.6)	
Divorced: *n* (%)	0 (0)	0 (0)	
Socio-economic status			
Low: *n* (%)	4 (25.0)	2 (11.1)	0.50
Lower-middle: *n* (%)	2 (12.5)	4 (22.2)	
Middle: *n* (%)	10 (62.5)	12 (66.7)	
Employment			
Unemployed: *n* (%)	10 (62.5)	9 (50.0)	0.51
Employed: *n* (%)	6 (37.5)	9 (50.0)	
Number of prior inpatient psychiatric admisisons			
Nil: *n* (%)	10 (62.5)	9 (50.0)	0.07
Up to two: *n* (%)	0 (0)	5 (27.8)	
Three or more: *n* (%)	6 (37.5)	4 (22.2)	

Retention within the trial was very good. 27 of the randomized participants completed the study. Of the 16 randomized to the TAU group, 5 were lost to follow up. Only 2 participants dropped out of the 18 randomized to the CaPE group. These participants were also lost to follow up. Acceptance of the CaPE intervention was good with 89% of participants attending all 12 sessions. There was no significant difference between group patient satisfaction scores at baseline but satisfaction was much higher for patients in the CaPE intervention than TAU (ES: 1.41, *p* < 0.001) after 12 weeks. It is worth noting that the difference in patient satisfaction at 3 months, between those participants in the CaPE intervention and those receiving TAU, is partly caused by a decrease in the satisfaction scores for those in the TAU group (Mean difference = −3.7, SD = 4.3).

For psychoeducation outcomes and clinical outcomes, Table 3 presents the effect sizes and results of the statistical modelling. Knowledge and attitudes towards bipolar (BKAQ) showed a moderate to large effect size (standardized ES = 0.68; non-significant) with scores higher in CaPE than TAU. Medication adherence also improved in the CaPE group relative to TAU (ES = −0.81; *p* = 0.018). For clinical outcomes, patients in the CaPE intervention showed large improvements in manic and depressive symptoms in comparison to TAU, as measured by the YMRS (ES = −1.18, *p* < 0.001) and BDI scale (ES = −1.17, *p* < 0.001). Quality of life measures also showed large effect sizes for CaPE versus TAU (EQ-5D Index: ES = 0.88, *p* = 0.014; EQ-5D VAS; ES = 1.14, *p* < 0.001). For the EQ-5D index score, using weights for Thailand rather than the UK gave a very similar result, (ES: 0.88, *p* = 0.011) indicating that the treatment group difference is robust.

**Table 3:** Feasibility and clinical outcome measures with treatment differences at 3 months, group mean differences and standardized effect size (standardized by the outcome score standard deviation)

		Baseline		3 months		Change		Adjusted mean difference† (95% CI)^#^	Standardized effect size	Test statistic and *p* value
		*n*	mean (sd)	*n*	mean (sd)	*n*	mean (sd)			
Patient Satisfaction	TAU	16	17.6 (3.3)	11	13.2 (1.9)	11	−3.7 (4.3)	4.00 (2.28, 5.28)	1.41	*z* = 5.30 *p* < 0.001
	CaPE	18	15.8 (2.4)	16	16.6 (3.0)	16	1.4 (2.9)			
BKAQ score	TAU	16	1247 (129)	11	1188 (257)	11	−54 (271)	150 (−52, 322)	0.68	*z* = 1.58 *p* = 0.113
	CaPE	18	1325 (201)	16	1358 (164)	16	34 (231)			
MMAS-4 score	TAU	16	1.3 (1.7)	11	2.1 (1.5)	11	0.7 (1.8)	−1.22 (−2.18, −0.14)	−0.81	*z* = 2.37 *p* = 0.018
	CaPE	18	1.7 (1.7)	16	0.9 (1.4)	16	−0.5 (1.8)			
YMRS	TAU	16	6.5 (1.2)	11	10.0 (3.9)	11	3.4 (3.4)	−6.0 (−8.7, 3.7)	−1.18^‡^	*z* = 4.67 *p* < 0.001
	CaPE	18	7.2 (1.5)	16	5.1 (5.0)	16	−1.9 (4.2)			
BDI	TAU	16	17.8 (11.5)	11	19.6 (8.6)	11	1.3 (13.6)	−10.3 (−16.8, −4.5)	−1.17	*z* = 3.21 *p* = 0.001
	CaPE	18	17.1 (5.3)	16	9.5 (6.3)	16	−7.1 (9.1)			
EQ-5D Index (UK)	TAU	16	7.6 (2.1)	11	8.3 (1.8)	11	−0.1 (0.4)	0.24 (0.10, 0.50)	0.88	*z* = 2.47 *p* = 0.014
	CaPE	18	7.4 (1.8)	16	6.4 (1.3)	16	0.07 (0.19)			
EQ-5D VAS	TAU	16	60.6 (24.0)	11	48.6 (18.2)	11	−9.5 (29.6_	26.8 (12.2, 41.8)	1.14	*z* = 3.65 *p* < 0.001
	CaPE	18	56.8 (26.7)	16	75.6 (20.6)	16	16.7 (29.6)			

*BKAQ* Bipolar Knowledge and Attitudes Questionnaire, *MMAS-4* measure of medication adherence, *YMRS* Young Mania Rating Scale, *BDI* Beck’s Depression Inventory, *EQ-5D* EuroQol-5 dimensions; Index and Visual Analogue Scale

## Discussion

The results of this exploratory trial show that culturally adapted psychoeducation was an acceptable and feasible intervention in a low-resource setting. Furthermore, our study indicated substantial effect sizes for improvements in measures of knowledge and attitudes towards bipolar disorder and medication adherence, following 12 sessions of psychoeducation (CaPE) when compared to the TAU group. The CaPE group also reported large improvements in clinical outcomes YMRS and BDI scores, compared to treatment as usual as well as EQ-5D quality of life scores.

Psychological interventions in low- and middle-income countries, including Pakistan, are not readily available. We are not aware of any studies of psychological interventions for bipolar disorder in Pakistan or other parts of the Indian Subcontinent. However, recent studies from low-income countries have reported CBT to be an effective treatment for other psychiatric problems such as major depressive disorder, psychotic disorder, and self-harm (Chatterjee et al. 2009; Husain et al. 2014; Naeem et al. 2015a, b). Social and cultural factors influence perception of symptoms of mental illness and hence, engagement with services (Bhikha et al. 2012). Psychological interventions need to be tailored according to socio-cultural needs of patients. Evidence suggests that cultural adaptation of psychological intervention is crucial (Naeem et al. 2009). Previous systematic reviews of the efficacy of culturally adapted mental health interventions have found a moderately strong benefit of culturally adapted interventions (Griner and Smith 2006; Chowdhary et al. 2014). Studies have reported that interventions targeted to a specific cultural group were four times more effective than interventions provided to groups consisting of participants from a variety of cultural backgrounds (Griner and Smith 2006). Furthermore, interventions conducted in the participants’ native language (if other than English) were twice as effective as interventions conducted in English (Griner and Smith 2006).

To the best of our knowledge, this is the first trial in Pakistan to investigate the feasibility and efficacy of individual culturally adapted psychoeducation in the management of bipolar disorder. Previous studies in high-income countries indicate that individual PE can be effective in improving clinical outcomes (Bond and Anderson 2015; Salcedo et al. 2016). Similarly, feasibility studies of PE interventions for bipolar disorder have indicated that they are feasible and acceptable treatment options in high-income countries (Fiorillo et al. 2016; Poole et al. 2015). Our results are comparable to findings from studies in other low- and middle-income settings. A study in Iran showed that following individual psychoeducation for bipolar disorder given in 8 sessions, there was a decrease in the number of relapses, in the rates of hospitalization and improvement in medication compliance when compared to the control groups (Javadpour et al. 2013). However, a study of young adults (aged 18-29) with bipolar disorder, receiving 8 sessions of individual psychoeducation added to treatment as usual in Brazil, did not find any significant difference in depressive and manic symptom improvement nor in quality of life measures between intervention and control groups (Cardoso Tde et al. 2014). Another study conducted in Turkey reported that four-session individual psychoeducation did not significantly decrease the number of relapses although the intervention group had a lower number of relapses, lower number of multiple mood episodes, and a lower hospitalization rate (Gumus et al. 2015). These findings indicate that the number of sessions of psychoeducation may be an important factor in determining efficacy.

A fully powered and longer clinical trial will address certain limitations. The first of these is that the study was carried out in a single centre; thus our findings may not be generalizable to the rest of Pakistan or indeed the rest of the South Asian population. Second, as this was a pilot trial, our sample size was small and not powered to detect efficacy of the intervention. It is important to interpret the effect sizes relating to clinical outcomes cautiously since most outcome measures were self-report scales and may be at risk of recall bias, social desirability bias and errors in self-observation. Moreover, using TAU as a control is likely to inflate the effect sizes for the intervention. Future studies should consider using attention placebo controls such as unstructured social support or befriending to minimize the risk of bias. In order to determine reliable data regarding the clinical efficacy of CaPE, future trials should also match groups for potential confounding factors such as concomitant pharmacological treatment, duration of illness, and type of disorder (i.e., type I or type II). Sub-types of bipolar disorder differ in terms of illness severity, clinical and social outcomes, response to treatments, and personal disability, and these differences may have had an impact on our findings. Furthermore, as we excluded patients with a psychiatric co-morbidity, our findings are not generalizable to those bipolar patients with comorbidities. Finally, the period for evaluating the effectiveness of CaPE in the present study was only 3 months after randomization, and therefore, we are unable to comment on the long-term efficacy of the intervention .

## Conclusions

The intervention used in our study was empirically based and culturally relevant. The results of the study indicate that CaPE was acceptable and feasible with very low drop out rate. We used blind raters to prevent rater bias in the assessment of YMRS scores. The results are quite promising and strongly indicate that a larger scale randomized controlled trial of a culturally adapted psychoeducation intervention in bipolar disorder is warranted. In addition, the trial shows that such an intervention could potentially improve clinical outcomes and quality of life in Pakistani patients suffering from bipolar disorder. A study with a larger sample size and longer period of follow-up will be useful to determine the clinical and cost effectiveness of CaPE and will help with the planning of efficient and culturally appropriate interventions in Pakistan that could serve as a model for other low-income countries.

## Additional file

**Additional file 1.** Bipolar Knowledge and Attitudes Questionnaire (BKAQ)

## Abbreviations

BD: bipolar disorder
BDI: Beck’s depression inventory
BKAQ: bipolar knowledge and attitudes questionnaire
BPAD: bipolar affective disorder
CBT: cognitive behavioural therapy
CaPE: culturally adapted psychoeducation
DSM: diagnostic and statistical manual of mental disorders
ES: effect size
EQ-5D: EuroQoL quality of life scale
PE: psychoeducation
TAU: treatment-as-usual
VAS: Visual Analogue Scale
YMRS: Young Mania Rating Scale

## References

[CR1] Ahmer S, Faruqui RA, Aijaz A (2007). Psychiatric rating scales in Urdu: a systematic review. BMC Psychiatry.

[CR2] Beck AT, Ward CH, Mendelson M (1961). An inventory for measuring depression. Arch Gen Psychiatry.

[CR3] Bhikha AG, Farooq S, Chaudhry N (2012). A systematic review of explanatory models of illness for psychosis in developing countries. Int Rev Psychiatry.

[CR4] Bond K, Anderson IM (2015). Psychoeducation for relapse prevention in bipolar disorder: a systematic review of efficacy in randomized controlled trials. Bipolar Disord.

[CR5] Brooks R, EuroQol Group (1996). EuroQol: the current state of play. Health Policy.

[CR6] Cardoso Tde A, Farias Cde A, Mondin TC (2014). Brief psychoeducation for bipolar disorder: impact on quality of life in young adults in a 6-month follow-up of a randomized controlled trial. Psychiatry Res.

[CR7] Chatterjee S, Pillai A, Jain S (2009). Outcomes of people with psychotic disorders in a community-based rehabilitation programme in rural India. Br J Psychiatry.

[CR8] Chowdhary N, Jotheeswaran AT, Nadkarni A (2014). The methods and outcomes of cultural adaptations of psychological treatments for depressive disorders: a systematic review. Psychol Med.

[CR9] Colom F, Vieta E, Martinez-Aran A (2003). A randomized trial on the efficacy of group psychoeducation in the prophylaxis of recurrences in bipolar patients whose disease is in remission. Arch Gen Psychiatry.

[CR10] Fajutrao L, Locklear J, Priaulx J (2009). A systematic review of the evidence of the burden of bipolar disorder in Europe. Clin Pract Epidemiol Ment Health.

[CR11] Farooq S, Nazar Z, Akhtar J (2010). Effect of fasting during Ramadan on serum lithium level and mental state in bipolar affective disorder. Int Clin Psychopharmacol.

[CR12] Fiorillo A, Del Vecchio V, Luciano M (2016). Feasibility of a psychoeducational family intervention for people with bipolar I disorder and their relatives: results from an Italian real-world multicentre study. J Affect Disord.

[CR13] Gater R, Waheed W, Husain N (2010). Social intervention for British Pakistani women with depression: randomised controlled trial. Br J Psychiatry.

[CR16] Goodwin GM, Haddad PM, Ferrier IN (2016). Evidence-based guidelines for treating bipolar disorder: revised third edition recommendations from the British Association for Psychopharmacology. J Psychopharmacol.

[CR17] Griner D, Smith TB (2006). Culturally adapted mental health intervention: a meta-analytic review. Psychotherapy.

[CR18] Gumus F, Buzlu S, Cakir S (2015). Effectiveness of individual psychoeducation on recurrence in bipolar disorder; a controlled study. Arch Psychiatr Nurs.

[CR19] Hashmi SK, Afridi MB, Abbas K (2007). Factors associated with adherence to anti-hypertensive treatment in Pakistan. PLoS ONE.

[CR20] Husain N, Afsar S, Ara J (2014). Brief psychological intervention after self-harm: randomised controlled trial from Pakistan. Br J Psychiatry.

[CR21] Javadpour A, Hedayati A, Dehbozorgi G (2013). The impact of a simple individual psycho-education program on quality of life, rate of relapse and medication adherence in bipolar disorder patients. Asian J Psychiatry.

[CR22] Julious SA (2005). Sample size of 12 per group rule of thumb for a pilot study. Pharm Stat.

[CR24] Khan AA, Marwat SK, Noor MM, Fatima S (2015). Reliability and validity of Beck Depression Inventory among general population in Khyber Pakhtunkhwa, Pakistan. J Ayub Med Coll Abbottabad.

[CR26] Masood Y, Lovell K, Lunat F (2015). Group psychological intervention for postnatal depression: a nested qualitative study with British South Asian women. BMC Womens Health.

[CR27] Morisky DE, Green LW, Levine DM (1986). Concurrent and predictive validity of a self-reported measure of medication adherence. Med Care.

[CR28] Naeem F, Gobbi M, Ayub M (2009). University students’ views about compatibility of cognitive behaviour therapy (CBT) with their personal, social and religious values (a study from Pakistan). Mental Health Relig Cult.

[CR29] Naeem F, Gul M, Irfan M (2015). Brief culturally adapted CBT (CaCBT) for depression: a randomized controlled trial from Pakistan. J Affect Disord.

[CR30] Naeem F, Saeed S, Irfan M (2015). Brief culturally adapted CBT for psychosis (CaCBTp): a randomized controlled trial from a low income country. Schizophr Res.

[CR31] Naeem F, Phiri P, Munshi T (2015). Using cognitive behaviour therapy with South Asian Muslims: findings from the culturally sensitive CBT project. Int Rev Psychiatry.

[CR32] Oud M, Mayo-Wilson E, Braidwood R (2016). Psychological interventions for adults with bipolar disorder: systematic review and meta-analysis. Br J Psychiatry.

[CR34] Poole R, Smith D, Simpson S (2015). Patients’ perspectives of the feasibility, acceptability and impact of a group-based psychoeducation programme for bipolar disorder: a qualitative analysis. BMC Psychiatry.

[CR36] Sajatovic M, Valenstein M, Blow F (2007). Treatment adherence with lithium and anticonvulsant medications among patients with bipolar disorder. Psychiatr Serv.

[CR37] Sajatovic M, Valenstein M, Blow FC (2006). Treatment adherence with antipsychotic medications in bipolar disorder. Bipolar Disord.

[CR38] Salcedo S, Gold AK, Sheikh S (2016). Empirically supported psychosocial interventions for bipolar disorder: current state of the research. J Affect Disord.

[CR39] Scott J, Colom F, Vieta E (2007). A meta-analysis of relapse rates with adjunctive psychological therapies compared to usual psychiatric treatment for bipolar disorders. Int J Neuropsychopharmacol.

[CR40] Scott J, Colom F, Popova E (2009). Long-term mental health resource utilization and cost of care following group psychoeducation or unstructured group support for bipolar disorders: a cost- benefit analysis. J Clin Psychiatry.

[CR41] Sim J, Lewis M (2012). The size of a pilot study for a clinical trial should be calculated in relation to considerations of precision and efficiency. J Clin Epidemiol.

[CR42] Stafford N, Colom F (2013). Purpose and effectiveness of psychoeducation in patients with bipolar disorder in a bipolar clinic setting. Acta Psychiatr Scand Suppl.

[CR43] Thase ME, Sachs GS (2000). Bipolar depression: pharmacotherapy and related therapeutic strategies. Biol Psychiatry.

[CR44] Tohen M, Suppes T, Baker RW (2002). Olanzapine combined with mood stabilizers in prevention of recurrence in bipolar disorder: an 18-month study. Eur Neuropsychopharmacol.

[CR45] Vázquez GH, Holtzman JN, Lolich M (2015). Recurrence rates in bipolar disorder: systematic comparison of long-term prospective, naturalistic studies versus randomized controlled trials. Eur Neuropsychopharmacol.

[CR46] Vos T, Flaxman AD, Naghavi M (2012). Years lived with disability (YLDs) for 1160 sequelae of 289 diseases and injuries 1990–2010: a systematic analysis for the Global Burden of Disease Study 2010. Lancet.

[CR47] Waqas A, Zubair M, Ghulam H (2014). Public stigma associated with mental illnesses in Pakistani university students: a cross sectional survey. PeerJ.

[CR48] Wittchen HU, Jacobi F, Rehm J (2011). The size and burden of mental disorders and other disorders of the brain in Europe 2010. Eur Neuropsychopharmacol.

[CR49] Yatham LN, Kennedy SH, Parikh SV (2013). Canadian Network for Mood and Anxiety Treatments (CANMAT) and International Society for Bipolar Disorders (ISBD) collaborative update of CANMAT guidelines for the management of patients with bipolar disorder: update 2013. Bipolar Disord.

[CR50] Young RC, Biggs JT, Ziegler VE (1978). A rating scale for mania: reliability, validity and sensitivity. Br J Psychiatry.

