# Supplementary material for: Pilot study of a culturally adapted psychoeducation (CaPE) intervention for bipolar disorder in Pakistan
Source: Int J Bipolar Disord. 2017 Feb 11;5:3. doi: 10.1186/s40345-017-0074-8 (PMC5307423; doi:10.1186/s40345-017-0074-8)
Supplement: Supplementary file 1 — Additional file 1. Bipolar Knowledge and Attitudes Questionnaire (BKAQ) [file 40345_2017_74_MOESM1_ESM.doc]

# Bipolar Knowledge & attitude questionnaire

This questionnaire is designed to measure Knowledge & attitude to the management of Bipolar in general practice. For each statement please circle the answer that reflects your degree of agreement with the statement.

1. Biochemical abnormality is the basis of Bipolar disorder.
   100 75 50 25 0
   *Strongly agree Agree Neutral Disagree Strongly disagree*
2. It is difficult to know if patients are unhappy or have a Bipolar disorder needing treatment. 100 75 50 25 0
   *Strongly agree Agree Neutral Disagree Strongly disagree*
3. People with bipolar disorder have poor stamina deal with life problems . 100 75 50 25 0
   *Strongly agree Agree Neutral Disagree Strongly disagree*
4. Bipolar is a patient response which cannot be changed.
   100 75 50 25 0
   *Strongly agree Agree Neutral Disagree Strongly disagree*
5. Becoming Bipolar is part of being old.
   100 75 50 25 0
   *Strongly agree Agree Neutral Disagree Strongly disagree*
6. Bipolar patients who do not respond to what GP’s do.
   100 75 50 25 0
   *Strongly agree Agree Neutral Disagree Strongly disagree*
7. Psychotherapy tends to be unsuccessful with Bipolar patients.
   100 75 50 25 0
   *Strongly agree Agree Neutral Disagree Strongly disagree*
8. Bipolar patients needing medicines are better off with a psychiatrist than a GP. 100 75 50 25 0
   *Strongly agree Agree Neutral Disagree Strongly disagree*
9. Bipolar treatment in general practice usually produces a satisfactory result.
   100 75 50 25 0
   *Strongly agree Agree Neutral Disagree Strongly disagree*
10. If psychotherapy was freely available, it would be more beneficial than medicines for most patients.
    100 75 50 25 0
    *Strongly agree Agree Neutral Disagree Strongly disagree*

**11.** People with bipolar disorder act the way they do because they want to be different.

100 75 50 25 0
 St*rongly agree Agree Neutral Disagree Strongly disagree*

**12.** Bipolar disorder is a defense mechanism displayed by people who cannot cope with everyday life

100 75 50 25 0
 *Strongly agree Agree Neutral Disagree Strongly disagree*

**13.** Bipolar disorder is caused by repressed feelings and emotions in the subconscious

100 75 50 25 0
 *Strongly agree Agree Neutral Disagree Strongly disagree*

**14.** Bipolar disorder can be caused by childhood emotional trauma

100 75 50 25 0
 *Strongly agree Agree Neutral Disagree Strongly disagree*

**15.** Bipolar disorder is caused by parents bringing up their children incorrectly

100 75 50 25 0
 *Strongly agree Agree Neutral Disagree Strongly disagree*

**16.** Bipolar disorder can be caused by being brought up by others with the disorder

100 75 50 25 0
 *Strongly agree Agree Neutral Disagree Strongly disagree*

**17.** Bipolar disorder can be brought on by taking drugs

100 75 50 25 0
 *Strongly agree Agree Neutral Disagree Strongly disagree*

**18.** Blood relatives of a manic depressive are likely to have other mental disorders

100 75 50 25 0
 *Strongly agree Agree Neutral Disagree Strongly disagree*

**19.** Bipolar disorder can be caused by inheriting the genes of someone with the disorder

100 75 50 25 0
 *Strongly agree Agree Neutral Disagree Strongly disagree*

**20.** Episodes of mania can be triggered by lack of sleep

100 75 50 25 0
 *Strongly agree Agree Neutral Disagree Strongly disagree*

**21.** Episodes of mania and depression have a purely biological basis (i.e. they are caused by chemical

imbalances in the brain)

100 75 50 25 0
*Strongly agree Agree Neutral Disagree Strongly disagree*

**22.** People with bipolar disorder who do not accept drug treatment or adhere to a drug regime prescribed by a doctor do not deserve any other help

100 75 50 25 0
*Strongly agree Agree Neutral Disagree Strongly disagree*

**23.** It is important to use ECT when patients are psychotic (out of touch with reality)

100 75 50 25 0
 *Strongly agree Agree Neutral Disagree Strongly disagree*

**24.** Drug treatment may not be accepted because of the possible side effects

100 75 50 25 0
 *Strongly agree Agree Neutral Disagree Strongly disagree*

**25.** Depressive episodes can be treated by rigorous exercise

100 75 50 25 0
*Strongly agree Agree Neutral Disagree Strongly disagree*
